# The evolving landscape of large language models and non-large language models in health care

**DOI:** 10.1038/s44401-026-00076-1

**Published:** 2026-03-09

**Authors:** Rui Yang, Huitao Li, Matthew Yu Heng Wong, Yuhe Ke, Xin Li, Kunyu Yu, Jingchi Liao, Jonathan Chong Kai Liew, Sabarinath Vinod Nair, Jasmine Chiat Ling Ong, Irene Li, Douglas Teodoro, Chuan Hong, Yifan Peng, Daniel Shu Wei Ting, Nan Liu

**Affiliations:** 1 Center for Biomedical Data Science, Duke–NUS Medical School, Singapore, Singapore.; 2 Duke-NUS AI + Medical Sciences Initiative, Duke-NUS Medical School, Singapore, Singapore.; 3 School of Clinical Medicine, University of Cambridge, Cambridge, UK.; 4 Division of Anesthesiology and Perioperative Medicine, Singapore General Hospital, Singapore, Singapore.; 5 School of Chemistry, Chemical Engineering and Biotechnology, Nanyang Technological University, Singapore, Singapore.; 6 Division of Pharmacy, Singapore General Hospital, Singapore, Singapore.; 7 Graduate School of Engineering, The University of Tokyo, Tokyo, Japan.; 8 Department of Radiology and Medical Informatics, Faculty of Medicine, University of Geneva, Geneva, Switzerland.; 9 Department of Biostatistics and Bioinformatics, Duke School of Medicine, Durham, NC, USA.; 10 Duke Clinical Research Institute, Durham, NC, USA.; 11 Department of Population Health Sciences, Weill Cornell Medicine, New York, NY, USA.; 12 Singapore Eye Research Institute, Singapore National Eye Center, Singapore, Singapore.; 13 Byers Eye Institute, Stanford University, Stanford, CA, USA.; 14 Artificial Intelligence Office, Singapore Health Services, Singapore, Singapore.; 15 Programme in Health Services Research & Population Health, Duke–NUS Medical School, Singapore, Singapore.; 16 NUS Artificial Intelligence Institute, National University of Singapore, Singapore, Singapore.; 17 These authors contributed equally: Rui Yang, Huitao Li.

## Abstract

We analyzed 19,123 natural language processing-related studies to explore the differences in task distributions and application contexts between large language models (LLMs) and non-LLM methods in health care. Through topic modeling analysis, we found that LLMs demonstrate advantages in open-ended tasks, while non-LLM methods dominate in information extraction tasks. These findings highlight the complementary strengths of the two technical paradigms and provide reference for their integration strategies in future health care applications.

In health care, large volumes of unstructured textual data are generated daily, including clinical narratives and biomedical literature^1^. Efficiently extracting and structuring this information is crucial for improving health care quality, supporting clinical decision-making, and advancing research^1^. To achieve these goals, natural language processing (NLP) techniques have been widely applied to tasks such as event extraction, diagnostic coding, and clinical trial matching^2–4^. The field of health NLP has undergone significant evolution from rule-based systems to statistical learning methods, and more recently, to deep learning approaches. These technological advancements have significantly advanced the ability to structure medical information and have contributed substantially to knowledge discovery^2,3,5^.

The rise of large language models (LLMs) is fundamentally reshaping the field of NLP, demonstrating powerful capabilities in text understanding, generation, and reasoning^6–10^. Unlike previous methods, which typically depend on large amounts of annotated data and are tailored for narrowly defined tasks, LLMs leverage large-scale pre-training on diverse corpora, enabling strong generalization across a wide range of applications^6^. In health care, LLMs have shown extensive potential in numerous areas, including consultation, diagnosis, management, education, and research^11,12^.

The emergence of LLMs has introduced novel technological pathways to health NLP, significantly expanding the scope of possible applications. However, it remains unclear whether the research focus of LLM differs from that of non-LLM methods. To address this gap, we conducted topic modeling on 19,123 relevant studies from multiple databases to compare task distributions and application contexts across technological paradigms, aiming to map the current research landscape and inform future directions. Figure 1 illustrates the overall workflow of this study.

We retrieved a total of 44,609 studies from PubMed, Embase, Scopus, and Web of Science. After removing duplicates, 20,228 unique studies remained. We then excluded 1,105 records that did not meet the criteria and ultimately analyzed 19,123 studies. Among them, 4,295 were categorized as LLM-related studies, while the remaining 14,828 were categorized as non-LLM ones. The PRISMA flow diagram is provided in Supplementary Information A.

To explore the distribution of patterns of LLM and non-LLM studies within the semantic space, we embedded all studies using the “MedCPT Article Encoder” model^13^. The resulting embeddings were subsequently reduced to four dimensions using the Uniform Manifold Approximation and Projection (UMAP) algorithm^14^. As shown in Fig. 2, the density distributions for each dimension exhibit pronounced differences between the two groups, with nearly complete separation observed in Dimension 1. Notably, LLM-related studies exhibit a more concentrated distribution, whereas non-LLM ones are characterized by a broader, more dispersed pattern.

Subsequently, we utilized BERTopic^15^ to automatically generate an initial set of 40 topics from the included studies. The medical expert (Y.K.) then reviewed and consolidated similar topics, resulting in a final set of 28 distinct topics. The keywords corresponding to each topic are listed in Supplementary Information B, while the number and proportion of studies under each topic are provided in Supplementary Information C. The similarity map of the 28 topics is presented in Supplementary Information D. Figure 3 shows the proportional distribution of research on LLM versus non-LLM studies across these topics.

We observed that LLM-related studies were disproportionately in topics such as “Medical Education” (46.92%), “Mental Health & Psychology” (11.62%), “Medical Image Analysis” (7.10%), and “Text Summarization” (6.98%). These findings highlight the capacity of LLMs to generate open-ended content and perform cross-modal analysis. The significant proportion of studies focused on “Medical Education” suggests that LLMs may provide scalable solutions for knowledge delivery and dissemination, supporting more flexible and interactive training environments. For example, LLMs can be used to simulate clinical scenarios and guideline-based self-assessment, thereby enhancing the interactivity and personalization of medical education and bringing the learning experience closer to real-world clinical practice^16^.

In addition, the high proportion of studies on “Mental Health & Psychology” is consistent with the fact that tasks in this domain usually involve conversation, interaction, and subjective experience expression. LLMs, with strong conversational capabilities, can support more natural human–AI interaction, scenario simulation, and response generation, making them particularly suitable for tasks characterized by interpersonal communication and semantic openness^17^. The prominence of “Medical Image Analysis” reflects the growing trend of multimodal processing in medical AI. This trend introduces new technological approaches to diagnostic interpretation and reporting and facilitates the integration of visual and textual information^18^. The relatively high representation of “Text Summarization” highlights the potential of LLMs to enhance clinical workflows by condensing lengthy clinical notes, discharge summaries, and radiology reports into more concise and accessible formats. This application may reduce the documentation burden on clinicians and enhance the efficiency of information retrieval^19^.

In contrast, analysis of non-LLM studies revealed that 21.56% focused on “Electronic Health Record”, 12.29% on “Named Entity Recognition”, and 7.74% on “Ontology”. These findings are consistent with the traditional focus of NLP on structured information extraction and concept recognition, but also highlight the continued use of controlled concept representation. Such tasks typically require models with high degrees of controllability and precision—areas where conventional approaches continue to demonstrate a distinct advantage^18,20^.

This study reveals the differences in research focus between LLMs and non-LLM methods in health care, demonstrating their complementary advantages across various tasks and application scenarios. From a temporal perspective, LLM-related studies showed marked growth, increasing sharply from 757 in 2023 to 2787 in 2024, reflecting their rapid adoption. In contrast, while non-LLM studies maintained steady growth during the same period (from 2191 to 3140), their growth rate was relatively moderate. With the continued advancement of LLMs featuring stronger reasoning capabilities (such as GPT-5^21^, Gemini 2.5^22^, and DeepSeek-R1^9^), we anticipate increasing interest in their applications in clinical reasoning, decision support, and population health analytics. These developments are likely to broaden the impact and utility of LLMs in advancing medical research and health care practice.

At the practical level, researchers and institutions may gradually shift from an “either-or” paradigm to a more integrated approach: for example, employing LLMs for generation, interpretation, and interaction, while continuing to use traditional NLP methods for structured information extraction and standardized concept representation, thereby creating a complementary technical pipeline across the complete analytical workflow^23^. This understanding of hybrid strategies has important implications across multiple dimensions. Regarding AI policy development, regulatory frameworks should consider the characteristics and risks of both technologies, regulating LLMs’ capabilities and potential biases while ensuring the accuracy and traceability of traditional methods in critical tasks. In medical education and training curricula, programs should encompass both the fundamentals of traditional NLP techniques and practical LLM applications, enabling clinicians and researchers to understand the applicable scenarios for different methods and to flexibly select tools based on specific needs. Regarding health care system readiness, institutions need to establish appropriate technical infrastructure, data governance systems, and evaluation frameworks to support the responsible application of both technologies in clinical settings.

Despite their promising potential, the integration of LLMs into real-world clinical settings remains in its early stages^24^. They often struggle with limitations such as incomplete reasoning chains and insufficient explainability when facing complex clinical tasks, thereby restricting their applicability and reliability in clinical practice^19,25–27^. Furthermore, the ongoing enhancement of LLM capabilities has raised ethical concerns, particularly in areas such as privacy protection and bias control^28^. Beyond principled discussions, future work could further emphasize actionable technical and institutional strategies, such as audit-based performance and bias evaluation, transparency reporting mechanisms, and responsible data-sharing norms. Building controllable, transparent, and accountable medical AI systems will be essential to safeguard fairness and accessibility in global health care delivery^28–30^.

Meanwhile, this study has several limitations. First, our categorization of studies into “LLM” and “non-LLM” groups employed a keyword-based rule approach, which may introduce selection bias and systematic misclassification. This method may miss studies that do not use standard terminology, and it is difficult to accurately distinguish between studies that actively apply LLMs versus those that only mention LLMs, as well as hybrid studies that use multiple methods simultaneously. Although we implemented random sampling and manual verification to minimize errors, this issue warrants further improvement through more refined multi-label classification methods. Second, the generated topics exhibit some heterogeneity: some topics are more task-oriented, while others are more aligned with medical specialties. This heterogeneity reflects the diversity of the health NLP research landscape, but also indicates that topics are not strictly homogeneous categories. Therefore, the interpretation of topics still requires integration of semantic context and expert judgment to further enhance topic consistency and interpretability.

## Methods

### Search strategy

We conducted a systematic literature search using PubMed, Embase, Scopus, and Web of Science to identify relevant studies published prior to March 1, 2025. The search strategy we used included terms related to “natural language processing” and “large language model”. We limited our search to “Title/Abstract” and included only “Article” in “English” focused on “Human”. The detailed search strategy is outlined in Supplementary Information E.

### Data processing

After retrieving all studies from the databases, we implemented a data processing workflow to ensure the quality of the data. First, we performed initial deduplication using EndNote, followed by a secondary deduplication with Rayyan to eliminate all redundant studies. Next, we identified and removed studies without available abstracts. Although the publication type was restricted to “Article” during the search stage, we conducted an additional check to remove any misclassified entries. In addition, we performed an iterative topic modeling analysis of the research corpus and removed irrelevant studies through anomaly topic detection.

After data cleaning, we classified all studies into LLM-related and non-LLM-related categories by matching keywords in the study titles and abstracts. To ensure classification reliability, we randomly sampled 100 studies for manual verification. The verification was independently conducted by two researchers (R.Y. and H.L.) with backgrounds in medical NLP. Of the 100 studies, 98 studies were correctly categorized. The specific keyword list is provided in Supplementary Information F.

### Topic modeling

Topic modeling is an unsupervised or semi-supervised learning technique used to automatically extract latent topics from documents. In this study, we utilized the “BERTopic” package to perform topic modeling on the retrieved studies^15^. Specifically, we employed the “MedCPT Article Encoder” model, which was trained on 255 million query-article pairs from PubMed search logs, to generate embeddings^13^. Subsequently, we applied the UMAP algorithm to reduce the dimensionality of the embeddings, preserving semantic information while significantly reducing computational complexity^14^. Next, we utilized the Hierarchical Density-Based Spatial Clustering of Applications with Noise algorithm to cluster the reduced embeddings, automatically determining the number of topics and identifying clusters with similar semantics^14,31^. Finally, we generated topic representations and optimized them using GPT-4o^32^. A medical expert (Y.K.) further consolidated similar topics through the most representative keywords of each topic. Additionally, to verify the accuracy of the topic modeling results, we randomly selected 10 studies from each topic for manual inspection, confirming that the assigned topics were consistent with the corresponding study contents.

## Supplementary Material

Supplementary information

**Supplementary information** The online version contains supplementary material available at https://doi.org/10.1038/s44401-026-00076-1.

## Figures and Tables

**Fig. 1 |:**
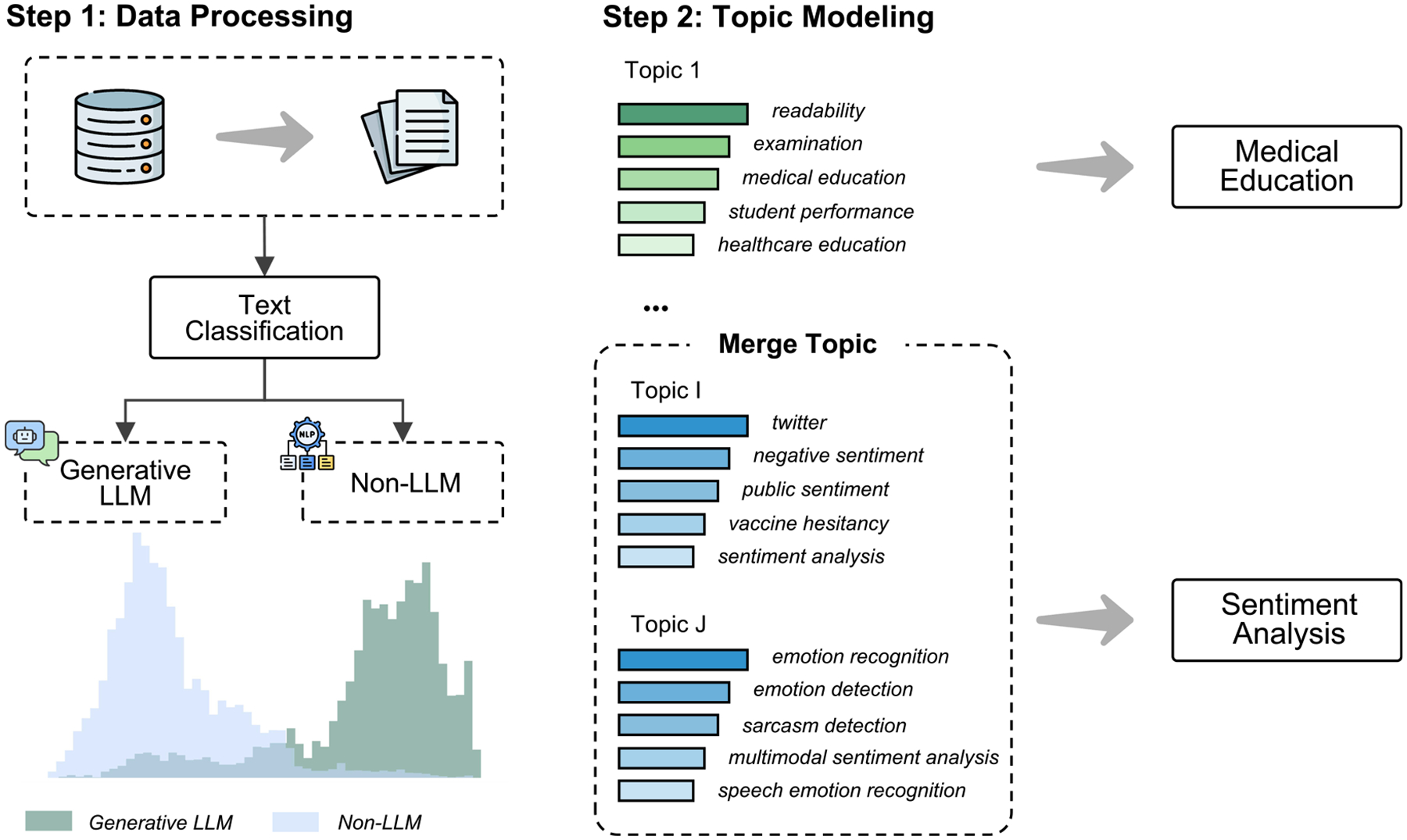
Study workflow overview. We retrieved 19,123 relevant studies from PubMed, Embase, Scopus, and Web of Science, and categorized them into LLM and non-LLM groups based on keywords. Subsequently, we performed topic modeling on all studies, and a medical expert reviewed and consolidated similar topics.

**Fig. 2 |:**
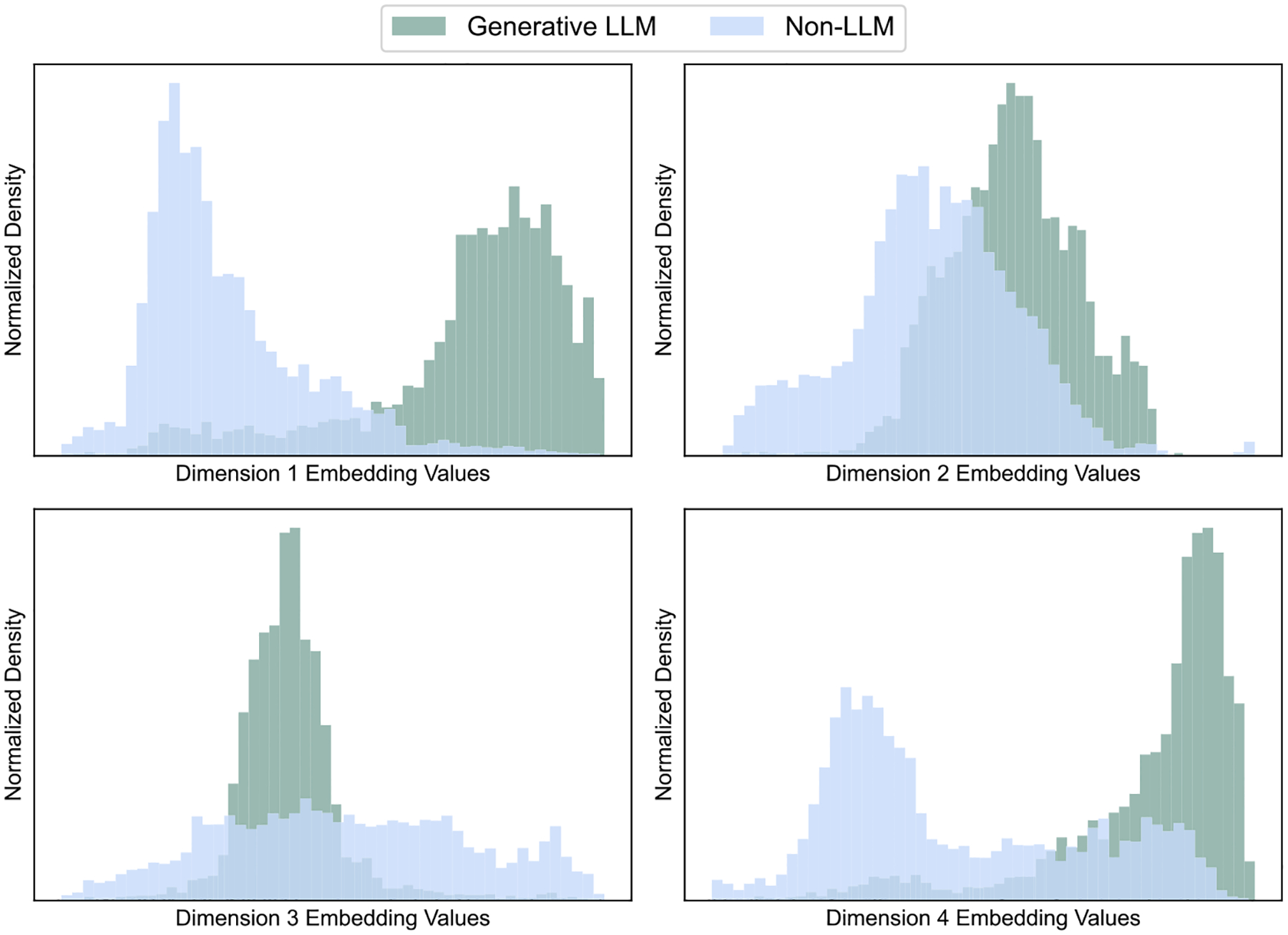
Semantic embeddings for LLM and Non-LLM studies. All studies were embedded using the “MedCPT Article Encoder” model and reduced to four dimensions via the UMAP algorithm.

**Fig. 3 |:**
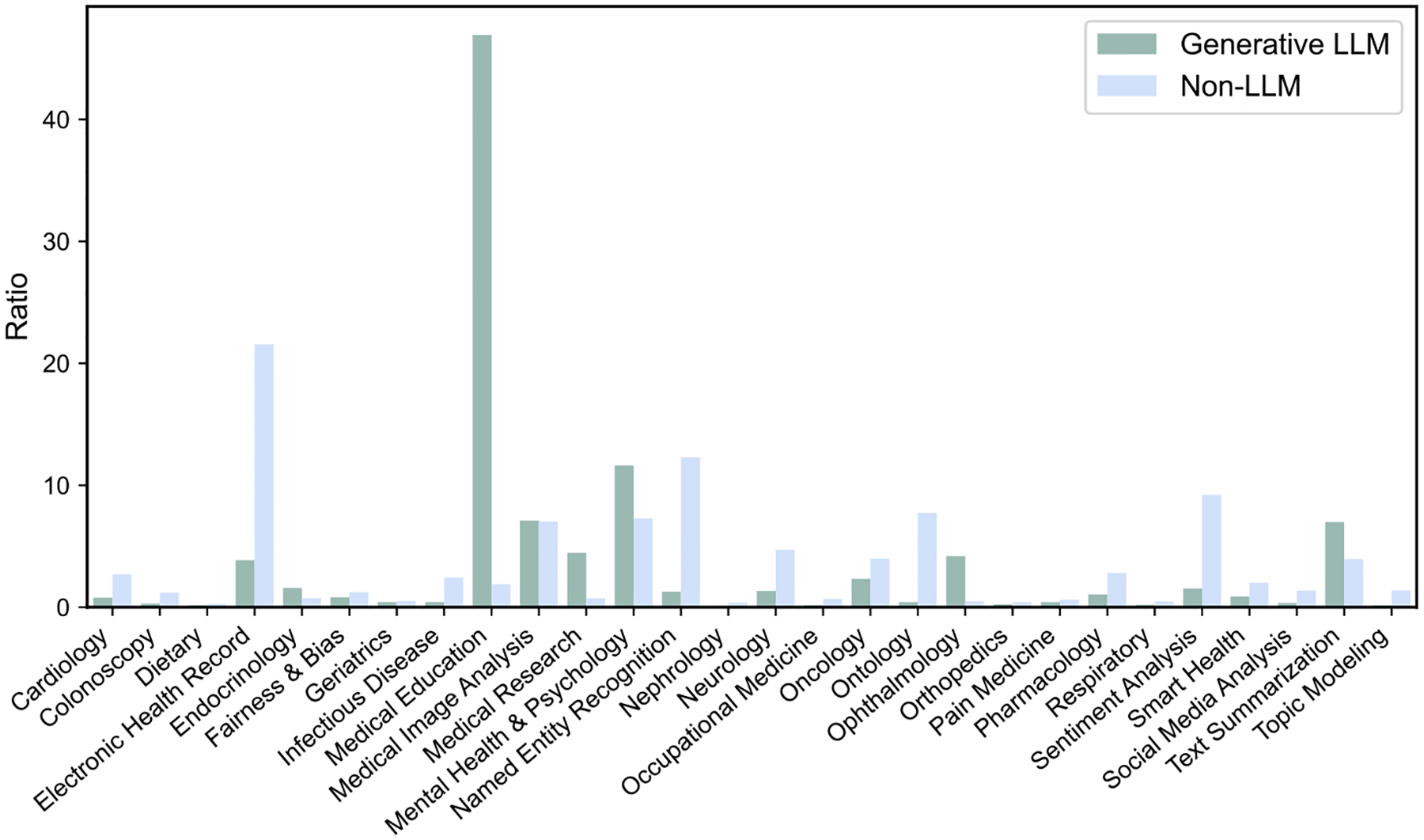
Distribution of topics across LLM and Non-LLM studies. LLMs exhibit higher concentrations in open-ended tasks such as “Medical Education”, “Mental Health & Psychology”, “Medical Image Analysis”, and “Text Summarization”, while non-LLM remains dominant in tasks such as “Electronic Health Record”, “Named Entity Recognition”, and “Ontology”.

## Data Availability

The data used in this study were obtained from subscription-based databases: PubMed, Embase, Scopus, and Web of Science. Access to these databases requires institutional or individual subscriptions. Due to licensing restrictions, the full data cannot be publicly shared. However, identifiers for the included studies will be provided upon reasonable request to researchers with appropriate access, and the full search strategy is available in the Supplementary Information to support reproducibility.

## Code availability

The code used in this study is available upon request.
