# Supplementary material for: The evolving landscape of large language models and non-large language models in health care
Source: Npj Health Syst. 2026 Mar 9;3:22. doi: 10.1038/s44401-026-00076-1 (PMC13038296; doi:10.1038/s44401-026-00076-1)
Supplement: Supplementary file 1 — Supplementary Information [file 44401_2026_76_MOESM1_ESM.pdf]

Supplementary Information A: PRISMA flow diagram

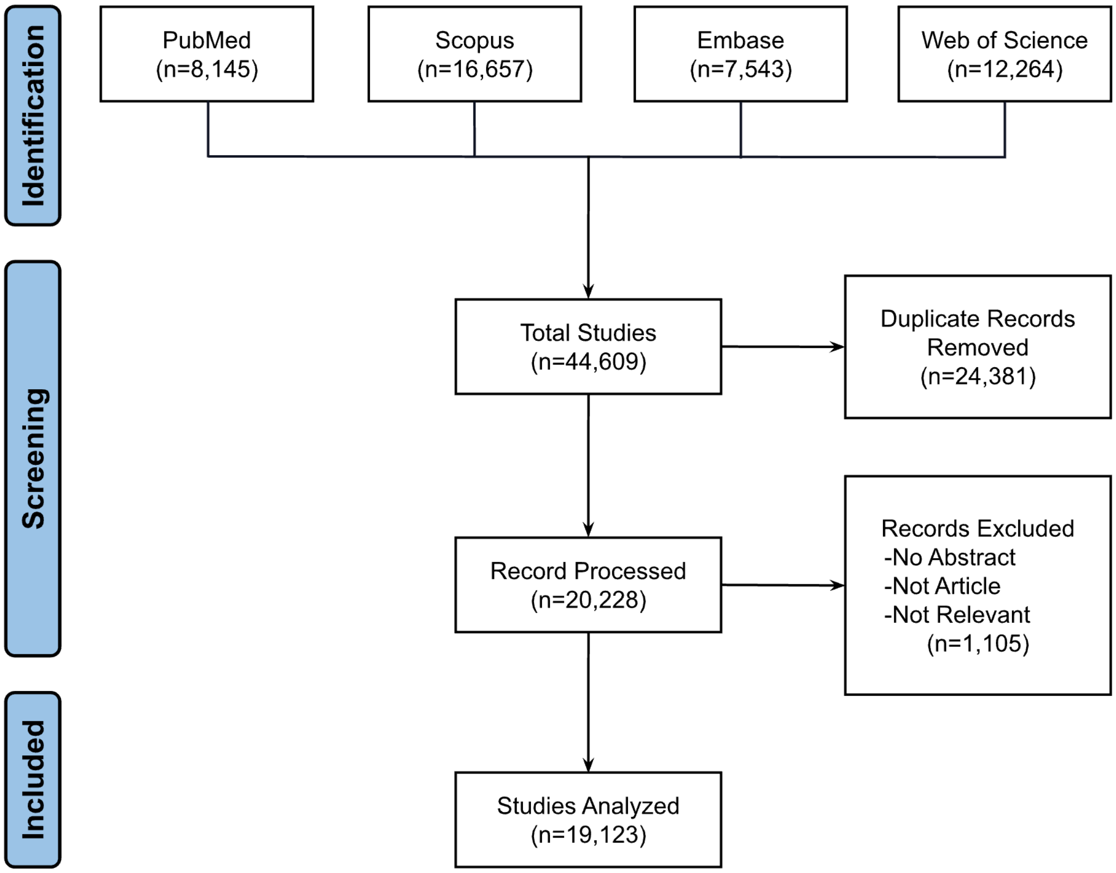

PRISMA flow diagram for identifying related studies. Our search retrieved 44,609 study records (n=8,145, 18.26% from PubMed; n=16,657, 37.34% from Scopus; n=7,543, 16.91% from Embase; n=12,264, 27.49% from Web of Science); of these, 20,228 (45.35%) were processed after deduplication (n=24,381, 54.65%). After excluding studies that lacked abstracts, were not articles, or were irrelevant (n=1,105, 5.46%), 19,123 (94.54%) records remained for analysis.

Supplementary Information B: Topic Keywords

ID	Keywords	Topic
1	clinical notes, electronic health records, patient information, annotation, unstructured data	Electronic Health Record
2	medical education, student performance, readability, examination, healthcare education	Medical Education
3	entity recognition, ner, information extraction, relation extraction, corpus	Named Entity Recognition
4	suicide prevention, depression detection, suicide attempts, suicidal ideation, suicide risk	Mental Health & Psychology
5	semantic similarity, semantic relatedness, sense disambiguation, biomedical ontologies	Ontology
6	question answering, biomedical summarization, large language models, text summarization, summarizer,	Text Summarization
7	radiology report, imaging, radiologists, diagnostic accuracy, error detection	Medical Image Analysis
8	image segmentation, medical image, convolutional neural network, attention, vision language	Medical Image Analysis
9	stroke, heart failure, ischemic stroke, ejection fraction, atrial fibrillation	Cardiology
10	mental health chatbot, conversational agent, mental health support, cognitive behavioral therapy, digital mental health	Mental Health & Psychology
11	public sentiment, twitter, negative sentiment, vaccine hesitancy, sentiment analysis	Sentiment Analysis
12	protein sequences, drug discovery, drug design, protein language models, molecular binding	Pharmacology

13	federated learning, security, adversarial attacks, IoT, edge devices	Smart Health
14	ophthalmology, glaucoma, retinal, ophthalmologist, diabetic retinopathy	Ophthalmology
15	precision medicine, liver metastases detection, cognitive computing, surgical innovations, predictive analytics	Oncology
16	social media, tweet, substance use, sentiment analysis, reddit	Sentiment Analysis
17	COVID-19, pneumonia, influenza, COPD, pandemic	Infectious Disease
18	dementia, alzheimer's disease, cognitive impairment, mild cognitive impairment, spontaneous speech	Neurology
19	scientific writing, medical writing, written abstract, ethics, AI content detection	Medical Research
20	colonoscopy, surveillance, colorectal cancer, adenoma, endoscopy	Colonoscopy
21	diabetes, diet, insulin, hypoglycemia, obesity, glucose	Endocrinology
22	eeg, electroencephalogram, eeg signals, sleep, brain computer	Neurology
23	aphasia, brain-computer interface, surprisal, inferior frontal gyrus, fMRI	Neurology
24	fake news, misinformation, hate speech, detection, social media	Social Media Analysis
25	patient review, review sentiment, physician review, sentiment score, sentiment analysis	Sentiment Analysis
26	topic modeling, latent dirichlet allocation, LDA, research trends, sustainable development goal	Topic Modeling

27	chemotherapy, radiation therapy, prostate cancer, chemoradiation, cancer, tumor	Oncology
28	stigmatizing language, black patient, white patient, judgment language, racial	Fairness & Bias
29	child speech, autism, communication, language development, autism spectrum disorder	Neurology
30	electronic health record, ehr, clinical documentation, ambient, secondary use	Electronic Health Record
31	epilepsy, epilepsy seizure, SUDEP, epilepsy surgery, functional seizures	Neurology
32	social robot, nursing robot, elderly, cognitive assistive, dementia	Geriatrics
33	pain management, headache disorder, migraine, chronic pain, complementary health approaches	Pain Medicine
34	hazard recognition, maintenance management, construction accident, safety management, accident category	Occupational Medicine
35	emotion recognition, multimodal sentiment analysis, emotion detection, speech emotion recognition, sarcasm detection	Sentiment Analysis
36	medical image retrieval, visual features, ImageCLEF, image search, image retrieval	Medical Image Analysis
37	asthma criteria, asthma ascertainment, childhood asthma, asthma care, asthma diagnosis	Respiratory
38	osteoporotic fracture, osteoporosis, bone health, fracture risk fracture radiology report	Orthopedics
39	food, recipe, ingredient, food safety, nutrition	Dietary
40	kidney failure, ckd, chronic kidney disease, proteinuria progression, proteinuria	Nephrology

Supplementary Information C: Comparison of Topic Distributions Between LLM and Non-LLM Studies

Topic	LLM	Non-LLM	Significance
Cardiology	33 (0.77%)	399 (2.69%)	***
Colonoscopy	13 (0.30%)	176 (1.19%)	***
Dietary	7 (0.16%)	43 (0.29%)	ns
Electronic Health Record	166 (3.86%)	3197 (21.56%)	***
Endocrinology	68 (1.58%)	109 (0.74%)	***
Fairness & Bias	35 (0.81%)	182 (1.23%)	ns
Geriatrics	18 (0.42%)	74 (0.50%)	ns
Infectious Disease	18 (0.42%)	360 (2.43%)	***
Medical Education	2015 (46.92%)	280 (1.89%)	***
Medical Image Analysis	305 (7.10%)	1041 (7.02%)	ns
Medical Research	192 (4.47%)	109 (0.74%)	***
Mental Health & Psychology	499 (11.62%)	1080 (7.28%)	***
Named Entity Recognition	55 (1.28%)	1823 (12.29%)	***
Nephrology	3 (0.07%)	56 (0.38%)	*
Neurology	57 (1.33%)	698 (4.71%)	***
Occupational Medicine	7 (0.16%)	101 (0.68%)	**
Oncology	100 (2.33%)	589 (3.97%)	***
Ontology	18 (0.42%)	1147 (7.74%)	***
Ophthalmology	180 (4.19%)	72 (0.49%)	***
Orthopedics	10 (0.23%)	63 (0.42%)	ns
Pain Medicine	18 (0.42%)	90 (0.61%)	ns

Pharmacology	45 (1.05%)	416 (2.81%)	***
Respiratory	8 (0.19%)	70 (0.47%)	ns
Sentiment Analysis	66 (1.54%)	1365 (9.21%)	***
Smart Health	38 (0.88%)	296 (2.00%)	***
Social Media Analysis	15 (0.35%)	203 (1.37%)	***
Text Summarization	300 (6.98%)	584 (3.94%)	***
Topic Modeling	6 (0.14%)	205 (1.38%)	***

The experiment compared the proportions of LLM and non-LLM studies across topics using a chi-square test of independence. P-values were adjusted for multiple comparisons using the Bonferroni correction. Statistical significance is indicated by asterisks (*$p < 0.05$, **$p < 0.01$, ***$p < 0.001$).

Supplementary Information D: Topic Similarity Map

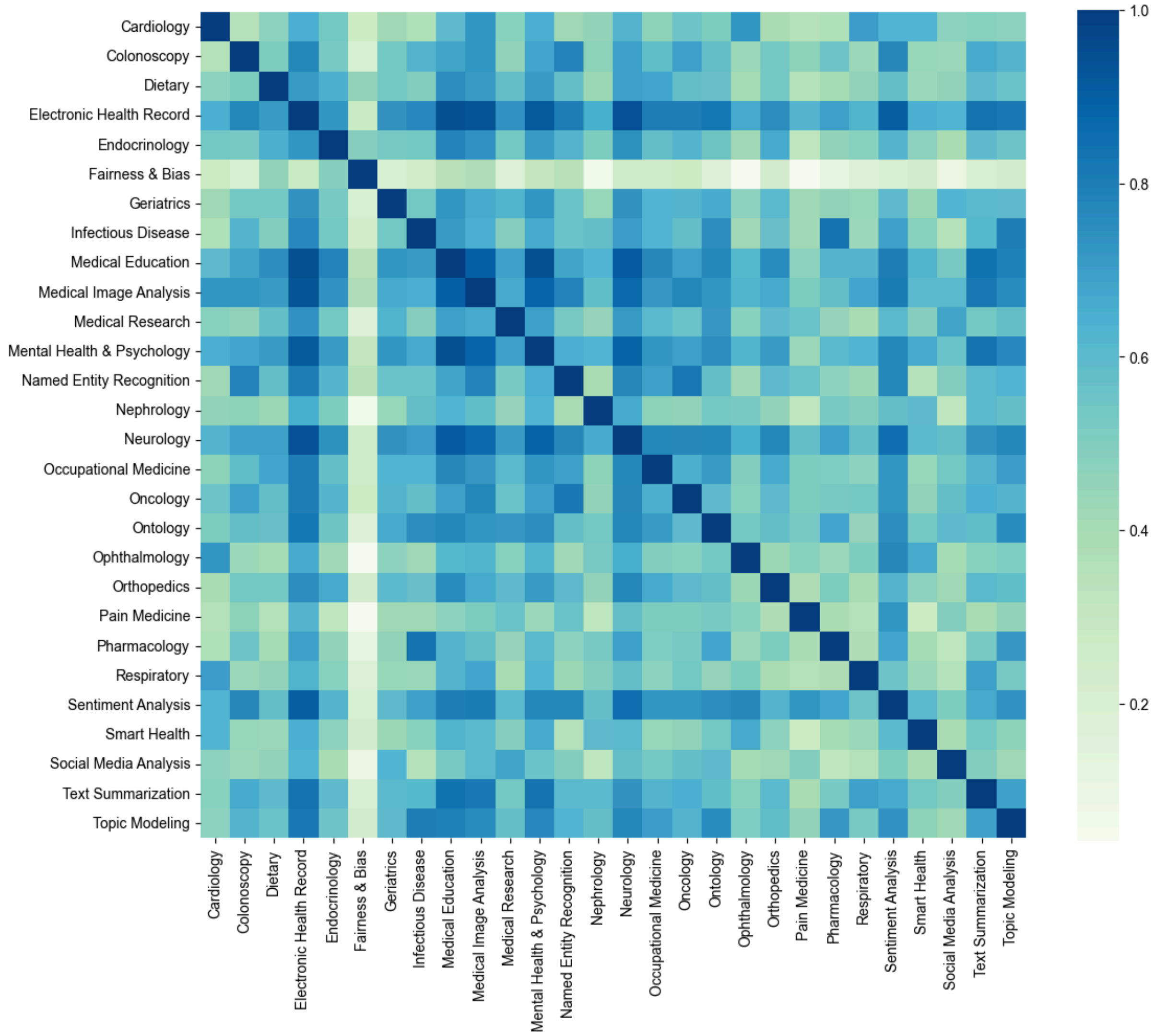

Supplementary Information E: Search Strategy

LLM and non-LLM

"natural language processing" OR "nlp" OR "language model*" OR "chatgpt" OR "llm" OR "bert" OR "retrieval-augmented generation" OR "retrieval augmented generation"

Medicine

"health*" OR "medic*" OR "biomedic*" OR "clinic*" OR "hospital" OR "patient*" OR "physician*" OR "doctor*"

PubMed: Search in **"Title/Abstract", "Journal Article", "English", "Human"**

Web of Science: Search in **"Topic"** and limit to **"Article", "English"**

Scopus: Search in **"TITLE-ABS-KEY"** and limit to **"Article", "English"**

Embase: Search in **"Title or Abstract"** and limit to **"Article", "English", "Humans"**

Supplementary Information F: LLM-related Keywords

Keyword	Regular Expression Used
LLaMA	\b(llama)\b
Med-PaLM	\b(med[-\s]?palm)\b
PaLM	\b(palm)\b
Med-Gemini_Gemini	\b(med[-\s]?gemini|gemini)\b
RAG	\b(rag|retrieval[-\s]?augmented[-\s]?generation)\b
LLM	\b(llm|large language model|chat[-\s]?gpt)\b
